# Development and testing of a 3D-printable polylactic acid device to optimize a water bioremediation process

**DOI:** 10.1186/s13568-020-01081-9

**Published:** 2020-08-15

**Authors:** Patricia Laura Marconi, Andrea Trentini, Myriam Zawoznik, Carlos Nadra, Juan Manuel Mercadé, Juan Gabriel Sánchez Novoa, Daniel Orozco, María Daniela Groppa

**Affiliations:** 1 CONICET, CEBBAD-Univ. Maimónides, Hidalgo 775, Buenos Aires, Argentina; 2 IQUIFIB, FFyB, Universidad de Buenos Aires, Junin 954, Buenos Aires, Argentina; 3 Universidad Nacional de Tres de Febrero, ACUMAR, Esmeralda 225, Buenos Aires, Argentina; 4 APRA-CIFA, Av. Castañares y Escalada s/n. Villa Soldati, Buenos Aires, Argentina; 5 CEBBAD, Univ. Maimónides, Hidalgo 775, Buenos Aires, Argentina; 6 IQUIFIB-CONICET, FFyB, Universidad de Buenos Aires, Junin 954, Buenos Aires, Argentina

**Keywords:** *Chlorella*, PLA, 3D-designed device, Matanza–Riachuelo watershed, Bioremediation, Bioprocess

## Abstract

In the present work, a remediation bioprocess based on the use of a native isolate of *Chlorella vulgaris* immobilized in an alginate matrix inside a polylactic acid (PLA) device is proposed. This microalga immobilized in alginate beads was previously shown to be useful for the reduction of several chemical and microbial contaminants present in the highly polluted water from the Matanza–Riachuelo watershed. However, these beads had a relatively short shelf life in the natural environment. To overcome this limitation, a 3D-printed PLA device was designed. PLA is a biocompatible and biodegradable material suitable for biotechnological applications. We used Erlenmeyers and stirred-tank bioreactors fed batch with Murashige Skoog (MS) culture medium or water from the Cildáñez stream (one of the water bodies of the aforementioned watershed) to estimate the growth kinetics parameters and the bioremediation capacity of immobilized-microalgal cells as an unconfined system (UcS) or a confined system (CfS) inside PLA devices on Cildáñez water. Although alga’s growth parameters were maximum in the UcS fed with MS medium as substrate, successful bioremediation of the target water was possible using the CfS: all inorganic nitrogen forms and total phosphorus were reduced at least by 90% after 5 days of bioprocess in an agitated bioreactor, whereas aerobic mesophilic bacteria decreased by about 85%. The number of coliforms also decreased. Standardized cytotoxicity tests using *Allium cepa* seeds carried out to prove the effectiveness of the bioremediation process, confirmed the high degree of decontamination achieved by the use of immobilized microalga confined in a 3D-printable PLA-device.

## Introduction

The anthropogenic impact on the Matanza–Riachuelo watershed (MRW) is critical and converts this area in one of the most polluted in Latin America (Guida-Johnson and Zuleta 2019). The Cildáñez stream is part of this watershed and presents a heavy contamination load, mainly composed of by-products of agriculture and waste materials derived from industries settled in the surroundings. Common ionic forms of dissolved inorganic nitrogen in aquatic ecosystems, including ammonium (NH_4_^+^), nitrite (NO_2_^−^), nitrate (NO_3_^−^), and phosphorus, as well as saprophytic and pathogenic bacteria from highly urbanized riversides, are the main Cildáñez stream contaminants (APRA 2016; ACUMAR 2017). It is essential to maximize research efforts directed to develop cleaning up technologies for this water body, close to which large populations are settled. In particular, reductions of nitrites and nitrates are of paramount significance, taking into account the serious health problems that these ions may generate, including methemoglobinemia and cancer (Ward et al. 2018; WHO 2011).

In a previous study, a native strain of the microalgae *Chlorella vulgaris* immobilized in alginate beads was tested and found to be a successful bioremediation agent (Trentini et al. 2017). Those results demonstrated the potential of this simple and cost-effective technology to remove several urban-water contaminants, offering as an additional advantage the possibility of microalga biomass recovery, which may serve as a source of third-generation biofuel. However, some field observations at the Cildáñez stream showed that alginate beads were degraded in a short time, limiting their applicability and efficiency (unpublished data).

In the present study, we describe a proof-of-concept assessment of an improved bioremediation technology based on the use of a 3D-printed device manufactured in an eco-friendly polymer: polylactic acid (PLA). This polymer allows gas diffusion and is stable at physiological temperatures and pH ranges, with the advantage of reduced weight and high mechanical strength resistance, among other interesting properties (Rosenzweig et al. 2015; Seol et al. 2018). Printing systems build 3D structures following a layer-by-layer approach, and PLA filaments are available in over 90 colors (Serra et al. 2014; Letcher and Waytachek 2014). 3D-PLA-printing filaments allow a versatile-design geometry and a customized architecture, able to fulfill specific biotechnological requirements such as those needed to improve bioremediation procedures. Here, we developed and tested a 3D-printed PLA device able to retain the alginate matrix in which alga cells can be immobilized and multiply, thus carrying out the target bioprocess of water decontamination, reaching high durability.

## Materials and methods

### Microorganisms and water samples

A unialgal culture of *C. vulgaris* strain LMPA-40 was obtained from the culture collection belonging to the Natural Science Faculty of the National University of Patagonia San Juan Bosco (Biological Data National System, SNDB-173). This culture was maintained on Murashige Skoog (MS) synthetic culture medium supplemented with sucrose (3% w/v) and indoleacetic acid (1 mg/L) as a growth regulator (Murashige and Skoog 1962) in Erlenmeyers of 250 mL containing 50 mL of culture media. Cultures were kept at 24 ± 2 °C in a shaker at 100 rpm, with mixotrophic conditions and a photoperiod of 16 h PAR (14,000 k, 400 μmol photon/m^2^/s).

Three water samples randomly obtained were collected from September 2017 to January 2018 from Cildáñez stream (34° 67′ 60.00″ S; 58° 44′ 37.06″ W) and analyzed in the laboratory of APRA (Agencia de Protección Ambiental, Ciudad Autónoma de Buenos Aires).

## 3D PLA-device design and characterization

**Fig. 1:**
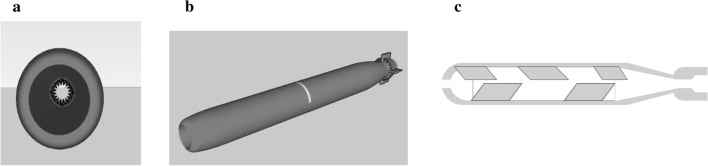
3D PLA prototype. Front view (**a**), external view (**b**), and longitudinal section (**c**)

The design and manufacture of the 3D-printed devices were made using specific software and a plotter (Crealty CR-10S, Shenzen Creality 3D Technol. Co., China). The polylactic acid (PLA) thread (diameter: 1.75 mm) was dispensed through a bronze needle (Volcano, internal diameter: 6 µm, at 220 °C); the plotting speed and the temperatures were 100 mm/seg and 55 °C, respectively. Figure 1 shows the device prototype, which had a torpedo shape opened at both ends. Five internal flaps to retain the alginate matrix and 5 external caudal flaps were included in the design. Size: 5 cm long, 1 cm internal diameter, basal aperture: 0.8 cm, apical aperture: 0.6 cm. The approximate volume and weight of the device were 3.93 cm^3^ and 0.738 g, respectively. The 3D device walls, of 4 mm thickness, were porous and rough. Devices were made in three colors: red, white, and translucent. Given the importance of light access through the PLA devices walls for the microalga growth, the transmittance (%T) of 8 × 8 mm fragments of the wall device was measured in a spectrophotometer Shimadzu UV1280 at different wavelengths. Transmittance (%T) was then calculated.

### Microalgae matrix preparation

Before use, the PLA devices were autoclaved at 0.1 MPa for 20 min in an equipment Chamberland (Arcano 80 L^®^). The process was repeated three times. A sterile alginate solution was mixed with a cellular suspension of *C. vulgaris* grown in MS medium with sucrose, at a rate of 2 × 10^6^ cells/mL of alginate. The mixture obtained was dropped on a stirred solution of CaCl_2_ (0.1 M) using a 50 mL syringe (8.9 mm diameter outlet). The beads thus obtained were incubated for 1 h and then washed with saline solution (NaCl 0.9% w/v). To prepare the PLA units, the mixture of alginate and *C. vulgaris* cells was injected into each device simultaneously with the cross-linking solution of CaCl_2_ (0.1 M) using peristaltic pumps (10 mL/min) with a cannula from each solution. The PLA devices thus prepared were incubated for 1 h and then washed with saline solution (NaCl 0.9% w/v) three times.

### Experimental design and measurements

Two bioremediation models were compared: *C. vulgaris* cells immobilized in alginate beads functioning as an unconfined system (UcS) versus immobilized *C. vulgaris* cells (same matrix) confined inside PLA devices (CfS). The synthetic culture medium MS and the target water from the Cildáñez stream (Cil.W) were used as substrates to compare the alga’s growth dynamics before defining the best bioremediation strategy. The assays were conducted as batch systems in 250 mL Erlenmeyers containing 50 mL of MS medium/Cildáñez water and 25 beads/5 PLA devices (obtained as described before) or in stirred-tank bioreactors (Minifors, Infors HT^®^, Switzerland) with a working volume of 1.5 L and an initial algal inoculum of 2% (w/v) contained in 100 beads/25 PLA devices. The bioreactor includes mechanical agitation achieved by a marine Rushton propeller (100 rpm) and a porous metal sparger to supply bubble aeration (0.5 vvm). The growth of *C. vulgaris* along the culture period was assessed by counting algae cells in a Neubauer chamber after dissolving the alginate matrix, as previously described (Trentini et al. 2017).

Once found the best conditions for algal growth, the bioprocess was scaled-up to be carried out in the bioreactors in the target water of the Cildáñez stream. The bioprocess lasted 5 days; the experimental units were kept at 24 ± 2 °C in autotrophic conditions, with a photoperiod of 16 h and the same PAR as that used for culture maintenance. Before and after each assay, the microalgal growth along time was assessed by counting *C. vulgaris* cells in a Neubauer chamber. The specific growth rate (µ) and the duplication time (dT) were calculated as described in Groppa et al. (2019). Water samples were filtered through a 0.45 μm membrane filter before physicochemical analysis. This analysis included pH, turbidity (NTU), electrical conductivity, nitrate, nitrite, ammonium, and total phosphorus determination. All analyses including microbial standard parameters (aerobic mesophyll bacteria and total coliforms) were performed according to the American Public Health Association (APHA) methods (2005) at the analytical service laboratory of APRA (Agencia de Protección Ambiental, Buenos Aires Ciudad). Heavy metals were also assessed by atomic absorption spectrometry (Analyst 800, Perkin Elmer, USA) using nitric acid-digested samples. Lead (Pb), cadmium (Cd), and arsenic (As) were determined using an electrothermal procedure (graphite oven). Chromium (Cr), copper (Cu), and zinc (Zn) were analyzed by flame spectrometry. Additionally, we calculated the denitrification rate (DR) as defined by Wang and Wu (2016):

$$ DR = \frac{{\left( {NO_{3}^{ - } + NO_{2}^{ - } } \right)i - \left( {NO_{3}^{ - } + NO_{2}^{ - } } \right)f  }}{HRT} $$

where (*NO*_*3*_^−^ + *NO*_*2*_^−^)_*i*_ are the initial nitrate and nitrite concentrations, (*NO*_*3*_^−^+* NO*_*2*_^−^)_*f*_ are the final nitrate and nitrite concentration (both in mg/L), and HRT is the hydraulic retention time of the bioprocess (5 day). The percentage of remediation was calculated as follows:

$$ \%  remediation = \frac{Ci - Cf}{Ci} \times 100 $$

where *Ci* and *Cf* are the initial and final contaminant concentrations, respectively, at the beginning and the end of the experiment.

### Cytotoxicity assays

Seeds of *Allium cepa* (about 300 seeds) were placed in Petri plates containing a filter paper imbibed with: i. 20 mL of water from the Cildáñez stream before the bioprocess (untreated water, UW), ii. 20 mL of water from the Cildáñez stream after the bioprocess (treated water, TW); and iii. 20 mL of distilled water (control, DW). The plates with the seeds were kept at 24 ± 2 °C for 48 h. Four days later, emerging seeds (uniform in size) were transferred to 200 cc-plastic pots containing perlite and went on growing by watering with the same water treatment. On day 6 from transplanting, 10 seedlings per treatment were removed from the pots to determine the mitotic index in isolated meristematic root cells (tip region) as follows: root tips were fixed for 24 h in acetic Carnoy, and chromosomes were stained with Orcein in 2% acetic acid (Basilico et al. 2017). The mitotic index (MI) was calculated by counting cells undergoing mitotic stages respect to the total number of cells observed using a microscope Olympus BX40 (Olympus ^®^). Finally, on day 12, all seedlings were harvested, and roots and shoots length was determined.

### Statistical analysis

Three independent experiments, including each four treatments (MS in Erlenmeyers, Cil.W in Erlenmeyers, MS in the bioreactor, Cil.W in the bioreactor), were carried out. Physicochemical and microbiological analyses were performed before and after the bioprocess (day 5), as already described. Experiments and analytical determinations were performed by triplicate. The cytotoxicity test was carried out using 100 onion seeds (10 independent experiments, 10 replicates each). The results were evaluated by ANOVA with post hoc Tukey test for multiple comparisons, or by Kruskal–Wallis test for non-normal variables, using Infostat software (Tukey 1953; Di Rienzo et al. 2013).

## Results

### Erlenmeyer experiments

**Fig. 2:**
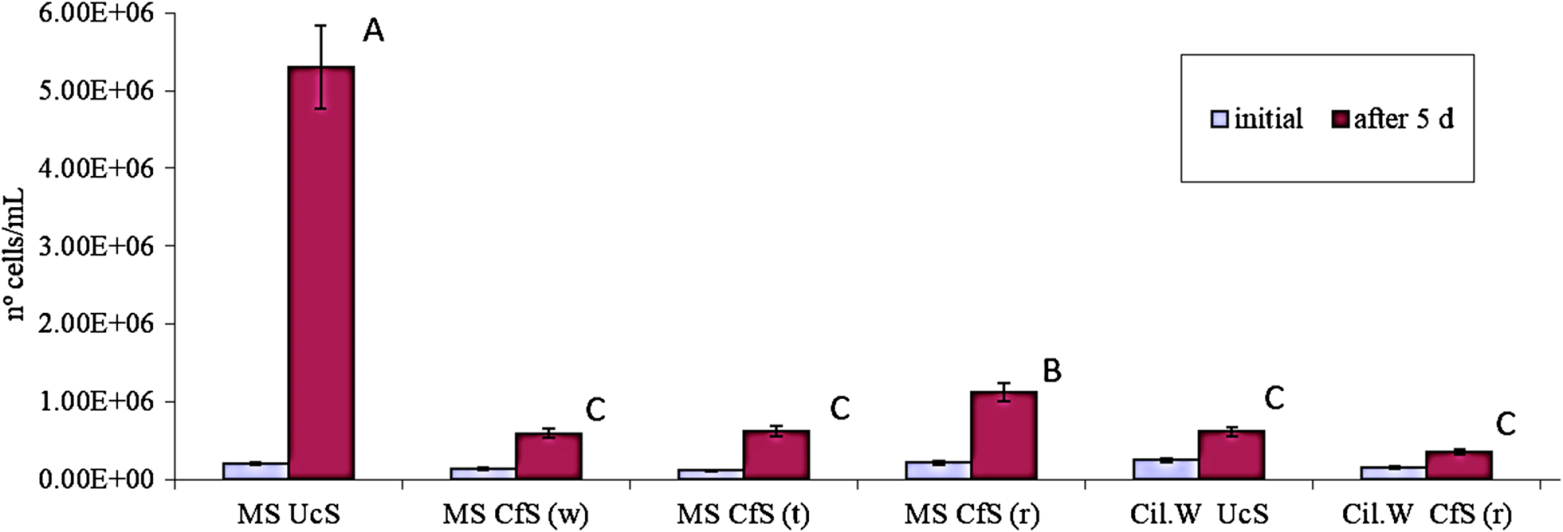
Cell number of *C. vulgaris* growing in Erlenmeyers. Initial and final cell numbers after a growth period of 5 days were determined as described in “Materials and methods”. *MS UcS* unconfined system in Murashigue Skoog (MS) growth medium, *MS CfS (w)* confined system in MS growth medium (white PLA device), *MS CfS (t)* confined system in MS growth medium (translucent PLA device), *MS CfS (r)* confined system in Cidáñez stream water (red PLA devices), *Cil.W UcS* unconfined system in Cidáñez stream water, *Cil.W CfS (r)* confined system in Cidáñez stream water (red PLA devices). Mean data from three independent experiments are shown. Letters indicate significant differences for the same substrate (p < 0.05)

As it may be noticed in Fig. 2, *C. vulgaris* cell numbers significantly increased after 5 days of cultivation when this microalga was entrapped in alginate beads and placed in MS culture medium as substrate, without any confinement. When the microalga cells were entrapped in the alginate matrix and confined inside the PLA scaffolds, algal growth was notoriously diminished. In addition, we verified that the red device allowed higher growth than the white one and the translucent one (which allowed similar alga growth). On the other hand, when we used water of the Cil.W to feed the system, the microalga number increased after 5 days but in a very limited extent (about 20% as compared to that observed in the synthetic culture medium), possibly due to the competence imposed by the presence of other microbial populations in this non-sterile substrate (Fig. 2).

We also estimated the number of *C. vulgaris* cells released from the alginate matrix in the systems under analysis and found no significant differences between them. A control treatment using distilled water demonstrated that microalgae could not multiply without the supply of inorganic nutrients, independently of its disposition (data not shown).

Based on these results, we focused on the red PLA device, which provides an acceptable microalga’s growth with the additional advantage of allowing an easier visualization inside the target substrate.

As the next step, we compared the growth kinetics of *C. vulgaris* cultivated as unconfined beads versus the confined system. These results are shown in Table 1. *C. vulgaris* cells achieved the highest growth rate (µ) and the lowest duplication time (dT) when cultivated as alginate beads in the synthetic medium without any physical confinement. When the alginate beads containing algae cells were incubated in the target substrate (Cil.W), the growth rate diminished by about 75%. On the other hand, it is important to note that in the target substrate, the difference between the unconfined system and the confined system becomes not significant.

**Table 1:** Growth kinetic parameters of *C. vulgaris* inferred from a 5 day-growth period in Erlenmeyers

	MS UcS	Cil.W UcS	MS CfS (r)	Cil.W CfS (r)
μ	0.672*	0.186	0.341	0.165
dT	1	3.7	2	4.2

Mean data from three independent experiments are shown. Asterisks indicate significant differences for the same substrate (p < 0.05)

*MS UcS* unconfined system in Murashige Skoog (MS) culture medium, *Cil.W UcS* unconfined system in Cidáñez stream water, *MS CfS (r)* confined system in MS culture medium (red PLA devices), *Cil.W CfS (r)* confined system in Cidáñez stream water (red PLA devices)

**Fig. 3:**
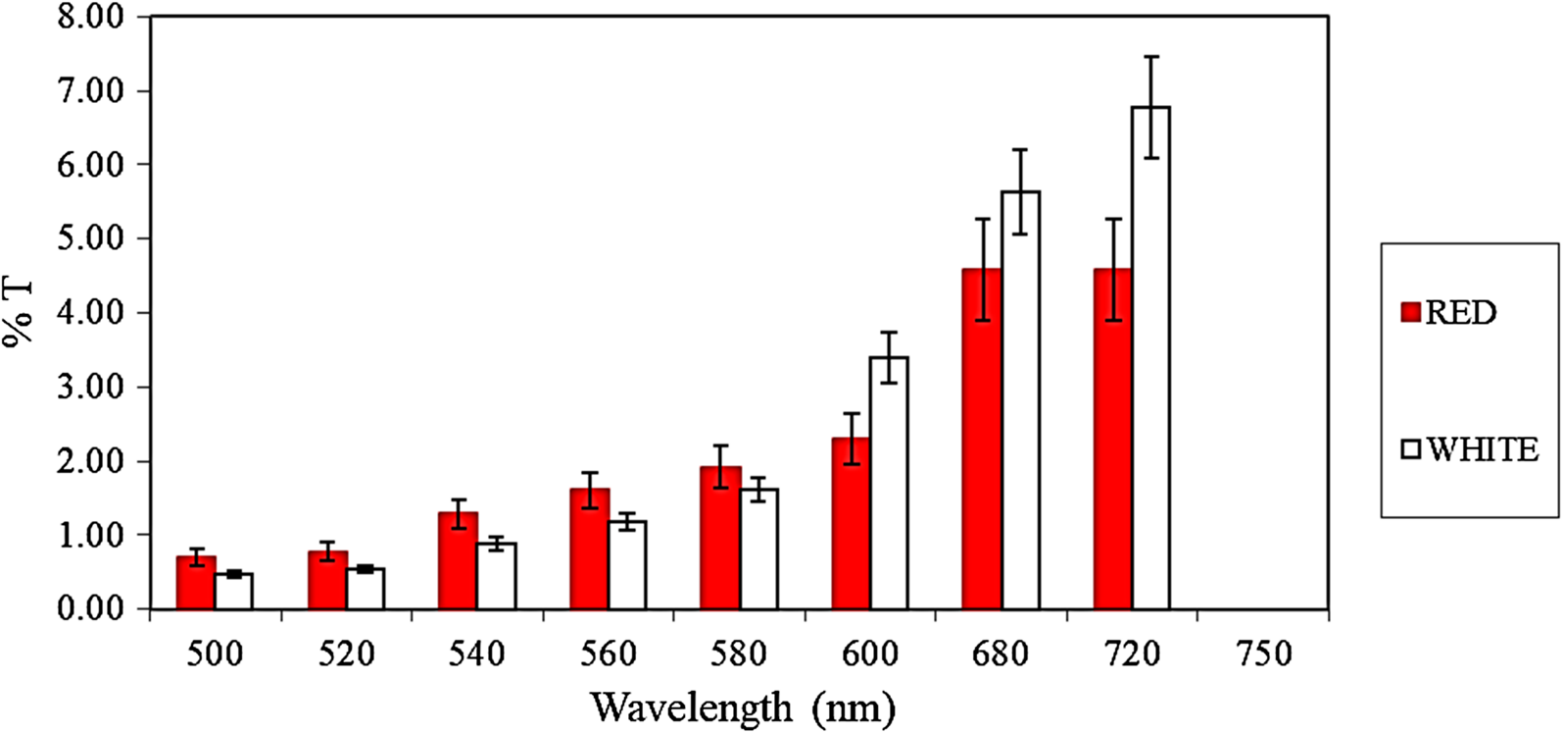
Transmittance (%) of the two PLA-devices. Mean data from three determinations are shown. Bars indicate standard deviations (SD)

Another important point to be considered is light access. Since our PLA-devices had opaque walls, photosynthetically active radiation (PAR) was expected to be a limiting factor. In this regard, we assessed the percentage of transmittance (%T) of the PLA walls manufactured in different colors at different wavelengths (Fig. 3).

It may be noticed that these values were very low irrespective of the color of the device, which corroborates the opacity of PLA and light availability inside the device as a limiting factor for alga growth. On the other hand, it is observed that the %T tend to increase at higher wavelengths, reaching the white device the maximum value at 720 nm and the red device the maximum values between 680 and 720 nm (similar %T, lower than the white one), coinciding with one of the absorption peaks of chlorophyll *a* and chlorophyll *b*. Also, it may be appreciated that at lower wavelengths (under 580 nm), the red PLA device has slightly higher transmittance than the white one.

The mechanical resistance of the PLA devices was checked by successive autoclaving cycles. We verified that these devices resisted a pressure of 0.11 MPa (15.95 psi) during 20 min, which is equivalent to 110 kN/m^2^ (11,200 kg/m^2^). Our devices did not undergo any deformation during the first and the second autoclaving cycles. After three autoclaving cycles, these devices tended to disaggregate, turning into a fine, crystalline sand-like stuff (data not shown).

### Bioreactor scaling up

To understand how the scale-up of the process would affect the growth parameters of *C. vulgaris*, we repeated the kinetics analysis based on cell numbers after 5 days of cultivation in aerated bioreactors, and added the use of the white device to select the best one for further bioremediation assessments; the results are shown in Table 2.

**Table 2:** Growth kinetic parameters of *C. vulgaris* inferred from a 5 day-growth period in bioreactors

	MS UcS	Cil.W UcS	MS CfS (r)	Cil.W CfS (r)	Cil.W CfS (w)
μ	0.363*	0.281	0.263	0.213	0.165
dT	1.9	2.5	2.6	3.3	4.2

Mean data from three independent experiments are shown. Asterisks indicate significant differences for the same substrate (p < 0.05)

*MS UcS* unconfined system in Murashige Skoog culture medium (MS), *Cil.W UcS* unconfined system in Cidáñez stream water, *MS CfS (r)* confined system (red PLA devices) in MS culture medium, *Cil.W CfS (r)* confined system in Cidáñez stream water (red PLA devices), *Cil.W CfS (w)* confined system in Cidáñez stream water (white PLA devices)

A significant reduction in the growth rate of the microalga in the unconfined system when the synthetic medium (MS) was used as the substrate was observed, resulting in a twofold increase in the duplication time as compared with the Erlenmeyer system (Tables 1 and 2). However, when Cildáñez water was used as the substrate, the opposite occurred: µ increased and dT decreased respect to the small-scale system. A similar trend was observed when alga cells were confined inside the red PLA devices: the scaling up tended to improve algal growth in the naturally-contaminated substrate and to reduce algal growth in the synthetic medium, reducing the gap between these two opposite environments for alga growth (ideal and real, respectively). Additionally, in this way, we corroborated that the white device was inferior to the red one regarding *C. vulgaris* growth, despite its higher transmittance at the most important wavelengths for microalga photosynthesis. For this reason, we chose the red device to evaluate the remediation effectiveness of the system proposed.

### Bioremediation performance of the system

As Table 3 shows, both systems (UcS and CfS; red PLA device) allowed significant reductions of over 90% in turbidity, and chemical parameters including nitrites, ammoniacal nitrogen, and total phosphorus contents.

**Table 3:** Water physicochemical and microbiological properties before and after the bioprocess

	Before treatment	After treatment		% remediation		Reference value^a^
		UcS	CfS	UcS	CfS	
pH	7.5	7.75	7.5			6–9
Turbidity (NTU)	110	44.5	40			
Nitrites (mg/L)	1.1	0.03	0.05	97.3	95.5	10
Ammoniacal nitrogen (mg/L)	11	0.099	0.2	99.1	98.2	1.5
Nitrates (mg/L)	16	10.5	0.9	34.4	94.4	< 10
Lead (µg/L)	14	ND	10	100	28.6	50
Total phosphorus (µg/L)	390	0.95	24	99.8	93.8	100
Denitrification rate		1095	2692			
Aerobic mesophyll bacteria (CFU/L)	4,732,000	33,000	730,000	99	84	1,000,000
Total coliforms (CFU/100 mL)	1,141,000	20	360,000	99	68	1000

^a^Reference values according to EPA 2011. *UD* undetectable. Remediation percentages were calculated as described in “Materials and methods”

We highlight the fact that in Cildáñez water, nitrates content before the bioprocess exceeded standard limits for drinking water proposed by international organizations such as WHO (11.3 mg/L) or US EPA (10 mg/L). Thus, this finding becomes highly significant. Despite the less alga growth, a higher reduction in nitrate contents was achieved with the CfS as compared to the UcS (94% and 34%, respectively). The pH remained constant during the assay in both treatments (Table 3). Another important finding was the reduction of phosphorus contents under both systems (UcS and CfS): over 93%.

Before the treatment, all tested metals except Pb were present in the water of the Cildañez stream in negligible amounts, far below those established as thresholds by the US EPA guidelines (2011) and close to the detection limit of our equipment. Lead content became undetectable after the UcS bioprocess, or it was reduced in the CfS treatment.

A very remarkable finding is the reduction in the number of aerobic mesophilic bacteria. Thus, the final microbial load with both systems was below the limit established by the US EPA (2011) (Table 3).

### Cytotoxicity of the treated waters

**Fig. 4:**
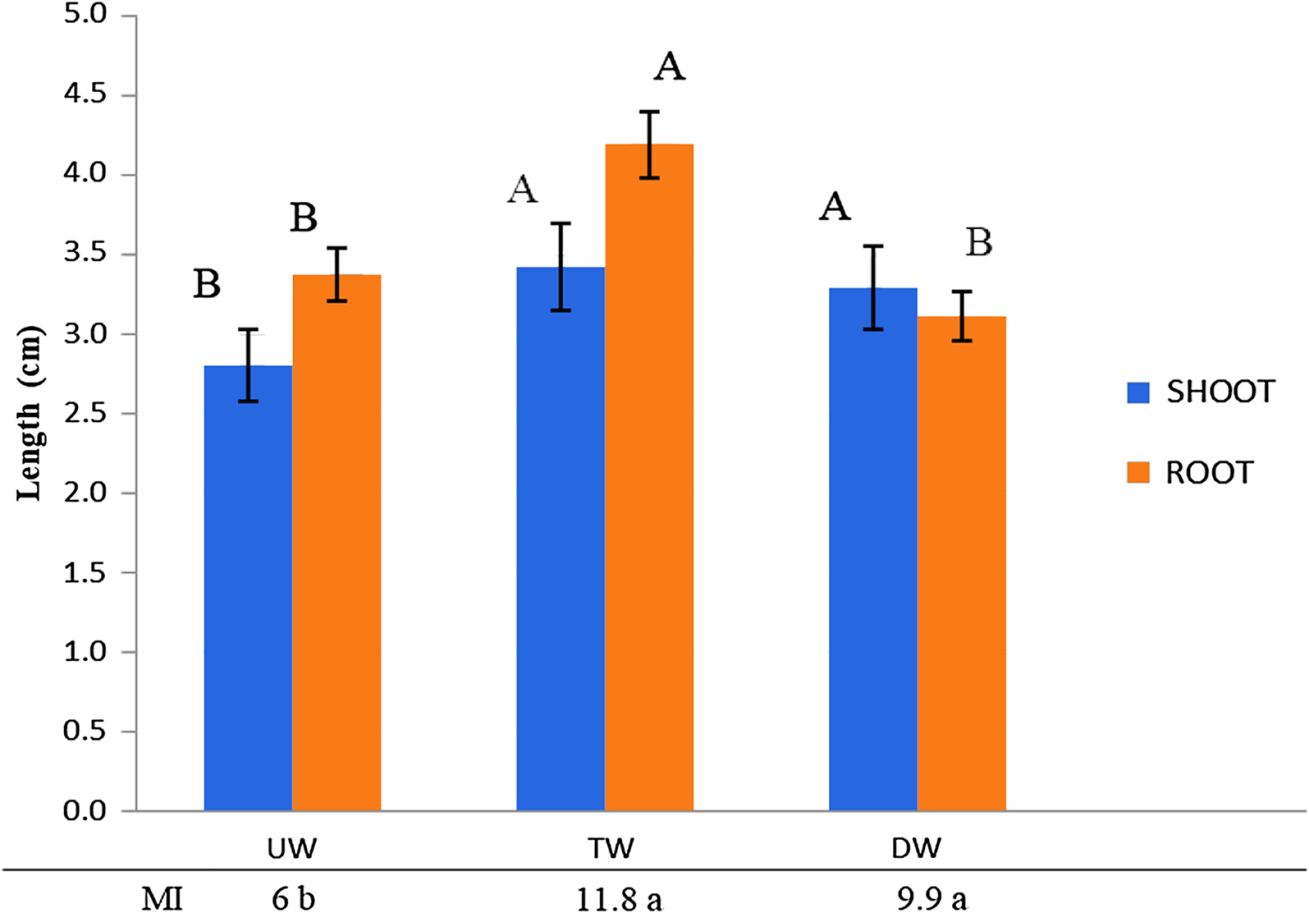
*A. cepa* root and shoot growth following seed imbibition with untreated or treated water from the Cildáñez stream. UW, untreated water: Cildáñez stream water before the bioprocess; TW, treated water: Cildáñez stream water after a 5-day bioprocess using the confined system (red PLA devices); DW: distilled water. MI: mitotic index of root tip cells. Measurements were performed as described in “Materials and methods”. Letters indicate significant differences for the same plant organ (p < 0.05)

Onion seeds were germinated using Cildáñez stream water before and after the bioprocess, and allow to grow under watering with the same water (untreated or treated). It may be noticed that the use of UW significantly reduced shoot and root length as compared to the use of TW or DW. In line with these findings, the MI of the epidermal cells in the root tip was significantly diminished in seedlings watered with UW (Fig. 4, bottom panel).

## Discussion

Several characteristics of PLA make this material particularly suitable to be used in specific fields such as medicine and cell, tissue, and organ engineering. PLA is a biodegradable and biocompatible material of relatively low cost and allows customized designs. For these reasons and trying to optimize a previously tested bioprocess based on the microalga *C. vulgaris*, we designed a 3D-printable PLA device, and we manufactured it in three different colors, to test their performances as confinement scaffolds for this microorganism in an optimal (MS) and a more realistic (Cildáñez stream water) environment. Despite the higher efficiency in terms of biomass production for immobilized algae not confined in the developed PLA devices, free alginate beads have reduced practical usefulness owing to the relatively short shelf life of this organic matrix in natural environments, where predators cannot be controlled. In this work, we designed and tested a 3D-printed polylactic acid device with a considerable mechanical resistance, where the alginate matrix entrapping *C. vulgaris* cells can be internally retained.

Synthetic culture media and water of Cildáñez stream (Cil.W) used to feed the system allowed microalga growth after 5 days in the confined or unconfined system. The highest growth rate (µ) and the lowest duplication time (dT) was achieved when algae were immobilized as alginate beads. One possible reason that would explain these differences regarding the algal growth rate is the shape of the alginate-immobilization matrix. In the case of the unconfined beads, the spherical shape is expected to allow greater gas diffusion than the cylindrical structures of the PLA devices, and therefore, better oxygenation. Besides, the higher nutrient availability due to the direct contact between the alga-trapping matrix and the substrate may account for this result. In this sense, it must be mentioned that even when the PLA device has semipermeable walls, diffused oxygen and nutrients may not achieve sufficient homogenization inside these devices to allow maximum algae growth.

It is well known that high nitrogen and phosphorous concentrations in water bodies promote the development of primary producers, and their excess ultimately results in the eutrophication of aquatic systems. In light of this scenario, it becomes essential to reduce the concentration of these solutes. The bioremediation performance of the combined system proposed shows a successful reduction in the physicochemical parameters measured. In line with this finding, the denitrification rate calculated was significantly higher in the CfS, representing a 2.5-fold increase as compared to the UcS. This finding is of particular relevance in the context of recent research, which highlights the usefulness of solid carbon sources to sustain the growth of heterotrophic denitrifying communities able to ultimately reduce nitrate ion to nitrogen gas (Schipper et al. 2010).

Our study was conceived as a basic proof of concept of an improved bioremediation approach based on the combined use of alginate-immobilized microalgae inside a solid, porous material, PLA, to eventually slow down the degradation rate of this biological system under natural conditions, not intended to discriminate the relative contribution of each component. Nevertheless, in line with previous results, we expected some effects of the PLA matrix itself, mainly related to adsorption phenomena. In this sense, it has been communicated that solid carbon substances may act as biofilm carriers (Chu and Wang 2013). Several biodegradable polymers such as poly-3-hydroxybutyric acid (PHB), polycaprolactone (PCL), polybutylene succinate, and polylactic acid have been developed and tested for solid-phase denitrification of eutrophized water bodies (Honda and Osawa 2002; Gibbs et al. 2004; Walters et al. 2009). More recently, Zhou et al. (2018) developed a multifunctional bionic three-dimensional polylactic acid-graphene oxide/chitosan sponge and proved its excellent removal efficiency for crystal violet. We hypothesize that the PLA device here designed and assessed may have favored the recruitment of denitrifying populations thriving in this water body due to its rough surface, allowing optimal nitrate reductase activity, as previously suggested (Wu et al. 2015).

Phosphorus consumption by microalgae is generally related to lipid metabolism and biomass accumulation. We verified a Pearson’s inverse correlation coefficient (ƿxy = − 0.75) between the remaining phosphorus levels in the substrate and the growth of algae, in line with previous reports (Liang et al. 2013). The results suggest that the increase in biomass could be accompanied by an increase in lipids, which is in agreement with an economy circuit in order to reuse, for example, in future biodiesel production.

Lead content became undetectable or reduced in UcS and CfS treatments, respectively. We ascribe this result to adsorption, a surface phenomenon that definitely favored the UcS: about 804 cm^2^ of total exposed area (100 beads; app. 8.04 cm^2^ each) versus approximately 91.85 cm^2^ of total exposed area in the CfS (25 devices; app. 3.7 cm^2^ each). It was suggested that heavy metal adsorption to *Chlorella* cell walls might be due to the presence of negative charges, mainly associated to amino, hydroxyl, carboxyl, acid, and sulfate functional groups, among others (Monteiro et al. 2012; Kaplan 2013; Ferraro et al. 2018; Sayadi et al. 2019). In addition, a recent study documents the usefulness of polylactic acid (PLA)/nano chitosan composite fibers to remove Cd^2+^ ions from water (Thomas et al. 2020).

Although these devices are nearly opaque, they allowed enough light and access to nutrients for algae growth to be possible. Therefore, the acquisition of biomass led to significant reductions in the content of various solutes that contaminated this water because they served as nutrients to support the growth of microalgae. Furthermore, microbial communities (including coliforms) were significantly reduced, probably as a consequence of the established competition between the introduced microalgae and the native microbial community. In this way, the *C. vulgaris* population could carry out an efficient remediation process, probably with the help of other native populations.

On the other hand, the presence of coliforms in a given natural watercourse reveals fecal contamination and therefore entails health risk. Of note is that also coliforms significantly decreased under both systems tested. However, given their high initial numbers, only the unconfined bioprocess could effectively reduce the number of these microorganisms to such an extent that acceptable levels according to standard guidelines could be achieved. Nevertheless, significant reductions were also observed with the confined system. We consider that competition for nutrients between the introduced alga, and these heterotrophic populations were the main driver of this phenomenon, even when specific antagonistic effects cannot be ruled out. PLA itself is not considered to exert antibacterial effects; however, several developments for medical purposes based on the addition of antibacterial substances (e.g., saccharides, metal ions) and nanofillers to PLA scaffolds have been published (Karakurt et al., 2019, and references therein).

Immobilized algae inside the PLA devices could bioremediate this heavily polluted stream with reductions of over 90% for key anions, including nitrate and nitrite, as well as phosphorus and potentially pathogenic bacteria such as coliforms. The use of the biodegradable PLA device could have favored the development of an active denitrifying community, which probably contributed to this N-load reduction. The efficiency of the bioremediation process proposed was corroborated through cytotoxicity tests using the well-known bioindicator plant *A. cepa*. These results are in line with the reduced GIs already reported (Groppa et al. 2019). Likewise, seeds imbibition with UW was associated with lower MIs, while the use of DW and TW resulted in higher values. According to Leme and Marin-Morales (2009), low MIs are typically linked to the adverse effects of xenobiotics on plant growth and development. The watering with TW led to a higher root length compared to UW and even also DW. This result may be ascribed to the presence of some root promoting substance or biological community in the treated water.

*Chlorella vulgaris* is a fast-growing microalga with minimal culture requirements and considerable resistance to stress conditions. This makes it a valuable tool to be used for optimized bioremediation processes.

## Data Availability

The datasets used during the current study are under CONICET guard and, also, they are available from the corresponding author on reasonable request.

## Abbreviations

PLA: Polylactic acid
MS: Murashige and Skoog
UcS: Unconfined system
CfS: Confined system
MRW: Matanza–Riachuelo watershed
%T: Transmittance
Cild.W: Cildáñez stream
NTU: Turbidity
DR: Denitrification rate
UW: Untreated water
TW: Treated water
DW: Distilled water
MI: Mitotic index
